# Useful Adjunct to the Emetin Method in the Treatment of Pyorrhea

**Published:** 1915-09

**Authors:** H. A. White

**Affiliations:** New Orleans, La., 1225 Maison Blanche Bldg.


### USEFUL ADJUNCT TO THE EMETIN METHOD IN THE TREATMENT OF PYORRHEA.

BY H. A. WHITE, D.D.S., NEW ORLEANS, LA.

For anyone to doubt the fact that the ameba is, at least, one of the causes of pyorrhea, in view of the work done on this subject by Drs. Barret and Smith, Chevaro, Bass and Johns of this city, and many others, does not seem possible. Yet this is the history of most great discoveries.

In a paper on this subject, published by Drs. Bass and Johns, they state, ''We have now examined two hundred cases of pyorrhea alveolaris, in all stages of the disease, and have found amebae present in all cases. We believe the conclusion is justified by our investigations, that amebae, chiefly, if not wholly, of the species entameba buccalis, are demonstrable by proper technic in the lesions of pyorrhea alveolaris in all stages of the disease, in all who have the disease in this locality.'' In a paper published later in the *Journal of the American Medical Association,* they state that, ''their studies now include more than three hundred cases.'' Surely, evidence of this character from men of national repute as bacteriologists should be convincing. The rapidity with which the amebae disappear and the disease shows improvement under the emetin treatment can be observed by any dentist, who will take the trouble to investigate the subject clinically.

I have treated to date more than twenty-five cases of pyorrhea with injections of emetin and am thoroughly convinced as to the success of the treatment. There is one phase in the treatment, however, that has heretofore been overlooked, and is a very important one. I refer to the extremely important part that adventitious organisms play in the disease. It has been stated by one writer that over one hundred varieties of bacteria have been found in the mouth, and another writer states, "The mouth even in health affords a great flora of pathogenic bacteria." It is possible in practically every case of pyorrhea to isolate staphylococci, streptococci, and other pathogenic bacteria, in profusion. When we remember that various systemic diseases, such as rheumatism, anemia, etc., often find their origin from mouth infection, due to the absorption of pathogenic bacteria or their products, we can realize the necessity of inhibiting the growth of these adventitious organisms whenever possible.

So, therefore, in the treatment and prevention of pyorrhea, as well as other diseases, which have a causative relation with the mouth, we should not lose sight of the importance of these secondary invaders and it should be our practice to combat their influence as much as possible. We can accomplish this, in a measure, by instructing our patients as to the proper methods of cleanliness and by repairing the damages in the mouth.

As to the influence an antiseptic medication has on the hastening of the cure of pyorrhea, I will cite one of my first cases, treated with emetin, as an example:

D. C., highly intelligent patient, has had pyorrhea three years. Has been treated by a number of dentists without any apparent benefit. Examination shows gum margins purple in color, gums tender, inflamed and spongy, bleed easily. Pus exuding from several pockets. The usual mechanical attention was given the teeth, consisting of scaling, etc., and emetin injections into the deltoid muscle and repeated according to Bass' and Johns' instructions. Patient improved rapidly and all pockets healed except two. Emetin in one-half of one per cent. solution was injected into these pockets daily, but they persistently refused to heal. I then injected into the pockets daily, and instructed the patient to use twice daily on his tooth brush, a 25 per cent. solution of tincture of ipecac and euthymol, which was supplied me by the Department of Experimental Medicine of Parke, Davis & Company. The next day the pockets were free from pus and showed signs of healing. In a few days, the pyorrhea was well.

Since treating this case, I have made use of the ipecac-euthymol solution as a direct injection into the pus pockets, as well as instructing my patients to use it, twice daily, on their tooth brush, in conjunction with injections of emetin, with the happiest results. Undoubtedly my patients have responded more quickly to treatment since the adoption of this adjunct therapeutic agent.

Euthymol is non-toxic and non-escharotic and, according to laboratory experiments, inhibits the growth of practically all pathogenic bacteria. While ipecac is powerfully amebaecidal, it is probably not antiseptic and, due to the mixed nature of the pyorrhea infection, it is very desirable to associate with it a mild bactericidal agent, such as the one mentioned.

1225 Maison Blanche Bldg.

